# Pre-existing structural control on the recent Holuhraun eruptions along the Bárðarbunga spreading center, Iceland

**DOI:** 10.1038/s41598-024-53790-x

**Published:** 2024-02-10

**Authors:** Arne Døssing, Mick Emil Kolster, Eduardo L. S. da Silva, Adrian R. Muxworthy, Jacob Thejll Petersen, Morten S. Riishuus

**Affiliations:** 1 Crustal Magnetometry Technology & Research Group (CMAGTRES), Division of Geomagnetism & Geospace, DTU Space, Centrifugevej 356, 2850 Kgs., Lyngby, Denmark; 2 UMag Solutions Aps, Nørgaardsvej 26, 2800 Lyngby, Denmark; 3 Department of Earth Science and Engineering, Imperial College London, South Kensington Campus, London, SW7 2AZ UK; 4 Department of Earth Sciences, University College London, Gower Street, London, WC1E 6BT UK; 5 Faroes Geological Survey, 34 Jóannesar Paturssonar gøta, Tórshavn, 100 Faroe Islands

**Keywords:** Geodynamics, Geology, Geomagnetism, Geophysics, Palaeomagnetism, Tectonics, Volcanology

## Abstract

The active rift zones in Iceland provide unique insight into the geodynamic processes of divergent plate boundaries. The geodynamics of Iceland are studied intensively, particularly, by geophysical methods sensitive to active and/or visible structures such as earthquake seismic and Synthetic Aperture Radar observations or aerial photographs. However, older and less active structures, that may exert a strong control on the presently active geodynamics, are often buried beneath recent volcanic or sedimentary deposits and are—due to their passive mode—overseen by the typical geophysical investigations. Aeromagnetic surveys provide spatial information about subsurface magnetization contrasts relating to both active and inactive structures. However, the aeromagnetic data in Iceland were collected in the 1970-80s and are relevant only to large-scale regional rift studies. With the availability of reliable drones and light-weight atomic scalar sensors, high-quality drone magnetic surveys can provide an unprecedented spatial resolution of both active and passive structures of rift systems as compared to conventional airborne surveys. Here, we present the results of a drone-towed magnetic scalar field and scalar gradiometry study of the north-northeast trending Bárðarbunga spreading center to the north of the Vatnajökull ice cap, Iceland. Our results provide new information about the structural complexity of rift zones with evidence of densely-spaced, conjugate and oblique faults throughout the area. Evidence is shown of a hitherto unknown and prominent east-northeast trending fault structure that coincides with the northern tip of the main eruption edifice of the 1797 and 2014-15 Holuhraun volcanic events. We suggest that this pre-existing structure controlled the locus of vertical magma migration during the two Holuhraun events.

## Introduction

**Figure 1:**
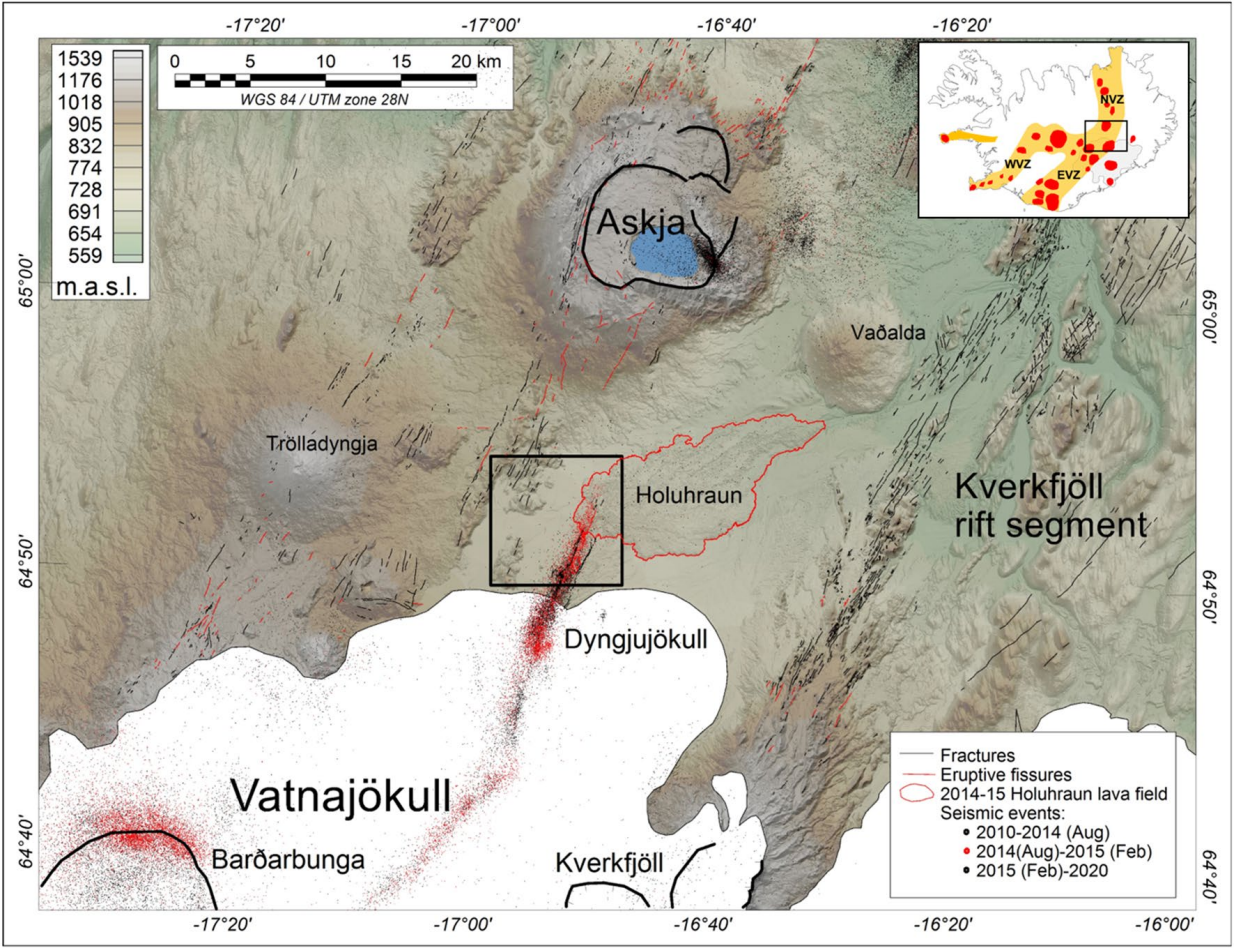
The Bárðarbunga-Askja volcanic systems of the Northern Volcanic Zone (NVZ), central Iceland. Black box: Area outlined in Fig. 2. Fractures and fissures^40^. Seismicity between 2010 and 2020 was obtained from http://hraun.vedur.is/ja/viku/. White areas: Vatnajökull ice sheet. Digital elevation model: https://ftp.lmi.is/gisdata/raster/. Inset map: Main volcanic spreading centres of Iceland. Abbreviations: EVZ, Eastern Volcanic Zone; NVZ, Northern Volcanic Zone; WVZ, Western Volcanic Zone.

The subaerial rift in Iceland constitutes the locus of plate separation between the Eurasian and North American plates, and the junction between seafloor spreading regimes of the North-East Atlantic and the Norwegian-Greenland Sea^1^. On a country-wide scale, the Iceland rift system can overall be split into the Western, Northern and Eastern Volcanic Zones (Fig. 1 - inset), which together comprise more than 30 partially overlapping regional spreading centers that are characterized mainly by a central volcano and a bisecting fissure swarm^2–5^.

The plate boundary of the Northern Volcanic Zone (NVZ) extends in the form of north-northeast ($\sim$N025^∘^) trending en echelon fissure swarms, starting beneath the Vatnajökull ice cap in central Iceland and extending towards the north coast^6,7^. The Askja and Bárðarbunga spreading centers constitute two of the major spreading centers of the NVZ (Fig. 1). The Bárðarbunga spreading center comprises a central volcano beneath the northwestern corner of the Vatnajökull ice cap^8,9^ and an associated bisecting fissure swarm that, in its northern section (the Dyngjuháls fissure swarm), extends beyond the edge of the ice cap^8^. The Dyngjuháls fissure swarm is divided into an $\sim$80-km-long north-trending swarm that extends to the west of the Askja volcano, and a shorter swarm extending to the northeast^10,11^. The latest eruption within the Bárðarbunga system took place along the northeastern segment in 2014-15, when a 48-km-long dike propagated laterally at several kilometers depth from the central volcano and erupted 13 days later at Holuhraun^12^. Here, it reoccupied the same eruptive vents and fractures as the 1797 Holuhraun eruption^10,11,13^. Over a time span of 6 months, a 500-m-long and 70-m-high spatter edifice built up along the principal eruptive fissure at its distal northeastern end and fed a lava field, reaching a volume of 1.6km^3^ and covering more than 84km^2^^14,15^.

**Figure 2:**
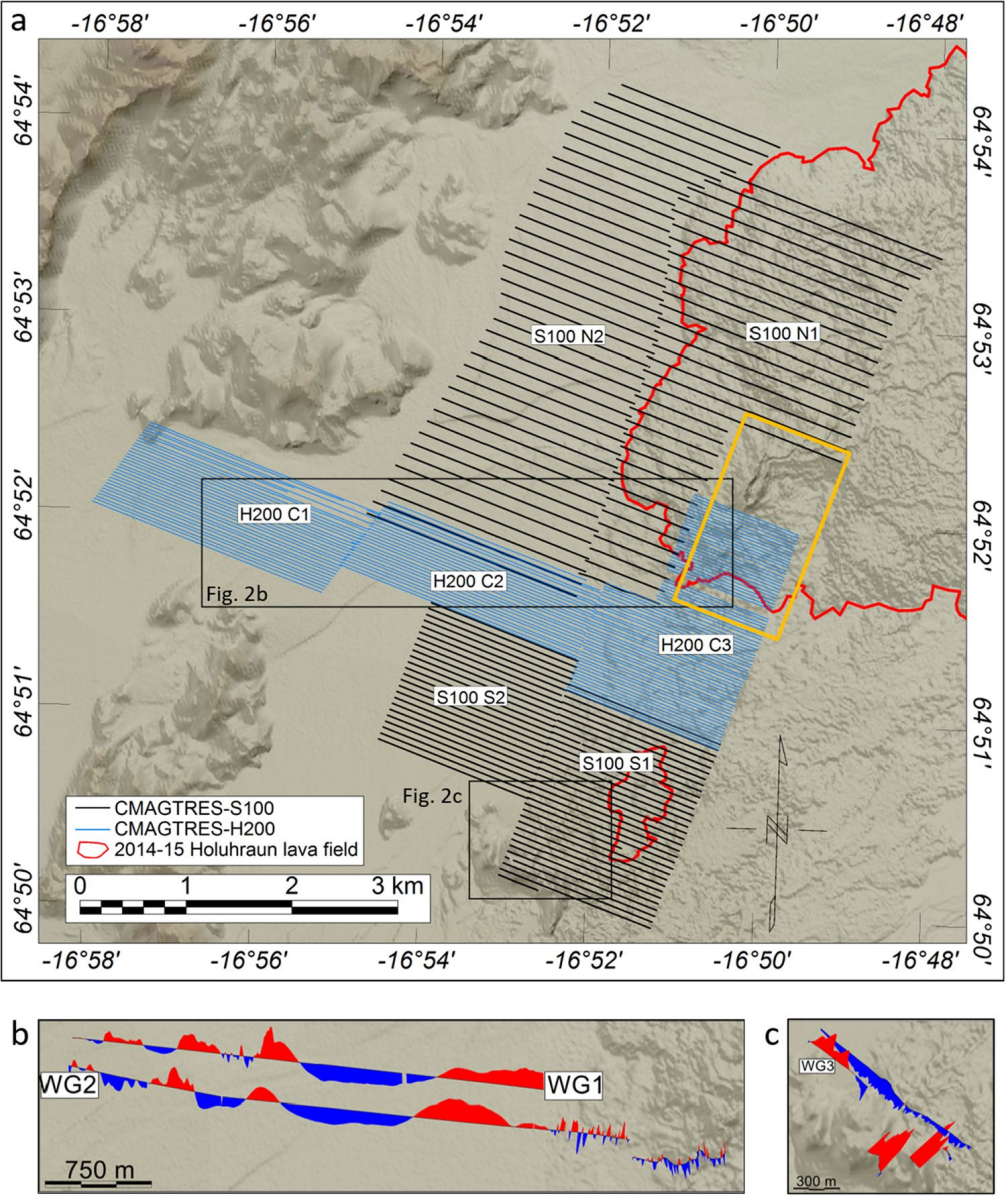
Overview of data collected. (**a**) S100 (black lines): CMAGTRES-S100 drone-towed single-sensor bird system. H200 (blue lines): CMAGTRES drone-towed transverse horizontal difference (gradiometry) bird system. The lines shown are trimmed survey lines. Black boxes: Areas outlined in (**b**) and (**c**). Orange box: Outline of drone photogrammetry data. Digital elevation model: https://ftp.lmi.is/gisdata/raster/. (**b**) WG1 and WG2 ground magnetic vertical residual profile data. (**c**) WG3 ground magnetic vertical residual profile data. Abbreviations: S100 N1-N2: S100 Northern Surveys 1-2; S100 S1-S2: S100 Southern Surveys 1-2; H200 C1-C3: H200 Central Surveys 1-3; WG1-WG3: Walking magnetic Gradiometry surveys.

**Figure 3:**
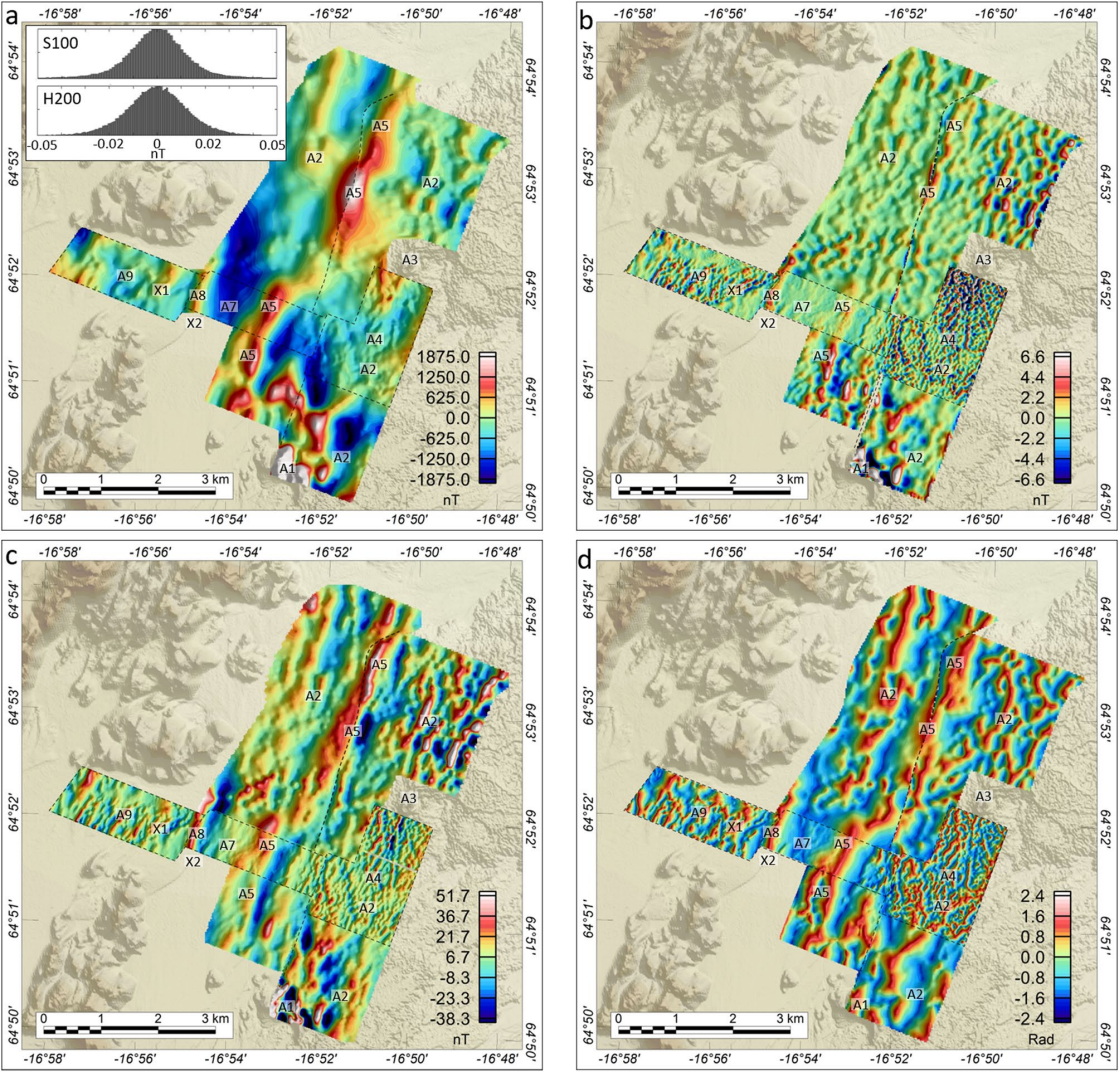
Total magnetic intensity (TMI) and derivative products. The magnetic data are displayed on top of digitized elevation data (available from https://ftp.lmi.is/gisdata/raster/). (**a**) TMI. Inset plots: Histogram plots of the estimated fourth-order difference noise levels of the S100 and H200 raw data, indicating a dynamic noise level of ±0.02nT for both systems during flight. (**b**) Discrete Vertical Difference of TMI (DVD). (**c**) Along-Track Difference of TMI (AD). (**d**) Tilt Derivative. Labels refer to anomalies and structures discussed in detail.

The Askja spreading centre is located about 25km north of the Vatnajökull ice cap and has a dominant fissure swarm that extends to the north. Despite the proximity of the Holuhraun lavas to the southern tip of the Askja volcanic system, analysis of major and trace element concentrations have demonstrated that Holuhraun was not sourced by lateral flow southward from Askja^11^.

The active rift zones processes in northern Iceland, including the Bárðarbunga system, are well-studied as they provide unique insight into the various geodynamic processes of divergent plate boundaries^12,16,17^. A significant part of the rift studies focus on monitoring, delineating, and understanding the active geodynamic processes using detailed earthquake seismic observations, geodetic observations, aerial photographs, and/or Synthetic Aperture Radar (SAR) images^10,12,18–20^. These methods are generally sensitive to structures and processes that are presently active, e.g., by detectable earthquakes, or at least visible at the surface (faults, fissures). However, to understand the many aspects of active geodynamic processes, it is critical to study also any hidden and potentially inactive structures, which may exert strong pre-existing structural control on the present-day dynamics^13,21–23^. Such structures may be concealed beneath sequences of young lava fields and/or glacial, fluvial and lacustrine sediments, in particular, in the areas surrounding the glaciers in Iceland, where detrital-loaded rivers and strong winds are continuously building up and re-surfacing large pro-glacial sedimentary plains^24,25^.

Magnetic survey investigations respond to subtle lateral contrasts in magnetic properties at depth^26–30^. Detailed airborne surveys with low-noise instrumentation are optimal for delineating minuscule magnetization contrasts at different depths and scales, and providing 2D spatial maps of both active and inactive structures. In areas like Iceland, the basaltic volcanic systems are expected to be dominated by linear magnetic anomalies, high in amplitude and short in wavelength, as a response to the linear structures (faults, fissures, intrusions) at - and beneath - the surface^31–33^. However, available airborne magnetic data in Iceland are generally unsuited for detailed structural investigations, being acquired in the 1970-80s, at nominal flight altitudes of 1200-2100m and with wide 3000m line spacing (http://www.lso.is/Magn-vefur/index.htm)^34,35^, i.e. anomaly wavelengths shorter than 6000m are in theory unresolved in the vintage data.

With the availability of reliable drone systems and lightweight quality atomic sensors, previously inaccessible areas or areas of low economic interest can now be investigated and even with superior data quality in terms of resolution and noise as compared to conventional airborne or ground magnetic surveys^36^. Here, we present a detailed drone and ground magnetic structural investigation of the ice-free highlands north of the Vatnajökull glacier, northeastern Iceland (Fig. 1); an area dominated by the active Bárðarbunga spreading center. Here, glacio-fluvial outwash, carried by the Jökulsá á Fjöllum river, prevents direct investigations of older, buried structures within the area. We carried out detailed drone magnetic investigations using two in-house developed high-sensitive single-sensor and double-sensor scalar magnetometer bird systems with the purpose of investigating pre-existing structural control on the 1797 and 2014-15 Holuhraun eruptive events and providing new magneto-structural insights into a subaerial spreading ridge at a level of detail not previously done. Our results provide new information about the structural complexity of rift zones in Iceland, showing detailed patterns of conjugate faults hidden beneath the glacial outwash plain. In addition, we show that the location of the principal eruptive fissure of the 1797 and 2014-15 Holuhraun eruptive events (Fig. 1) within the northeastern Bárðarbunga fissure swarm was guided by a strong, hitherto unknown, pre-existing structural control.

## Results

### Surveys

Seven drone magnetic surveys, totaling more than 500 line-km, and three ground magnetic surveys were collected during the campaign (Fig. 2; Suppl. Mat. Section S1). The central study area was covered by three high-resolution (30m line spacing, 30m altitude) scalar magnetic Transverse Horizontal Difference (THD) gradiometry surveys (named H200 C1–C3), bordered to the north and south by four medium-resolution (60-125m line spacing, 40-50m altitude) scalar-field surveys (named S100 N1–N2, S100 S1–S2) (Fig. 2). See Methods for details about the drone systems, survey specifications and data processing. In addition, a drone photogrammetry survey was collected across the 2014 Holuhraun eruption site (location in Fig. 2), and seven rock and one sedimentary sample were collected for rock magnetic analysis.

### TMI magnetic anomalies

The Total Magnetic Intensity data (TMI) ranges between -2500nT and 8000nT within the survey area (Fig. 3a), highest across the sub-angular topographic highs to the southeast (A1). In the remainder of the area, north-northeast trending ($\sim$N025^∘^) curvilinear-to-linear anomalies dominate the TMI. This trend is parallel to the regional topographic fabric (see Suppl. Mat. S2) and to the strike of the Northern Volcanic Zone (Fig. 1), but is 11^∘^ clockwise from the normal to the regional plate spreading direction of 104^∘^^37^. Many of the $\sim$N025^∘^ trending anomalies are traceable for distances of one kilometer or more.

The recent Holuhraun lavas in the eastern part of the survey area generate high-amplitude, short wavelength, curvilinear-to-linear anomalies (A2) with a dominating north-northeastern trend (Fig. 3a-d; see Suppl. Mat. S3 for interpreted magnetic lineaments). In the vicinity of the main Holuhraun eruption edifice (A3), the short-wavelength linear anomalies constitute a system of radiating lineaments (A4), indicating a radial stress field near the edifice during dyke emplacement. Away from the main edifice, the transition to parallel, linear anomalies reflects a gradual transition to a non-radial, regional, stress field. Similar observations radiating dyke swarms are found in relation to both small and giant volcanic systems^28,38,39^.

Medium wavelength anomalies dominate the TMI data across the Jökulsá á Fjöllum river glacio-fluvial outwash plain to the west of the Holuhraun lava field (Fig. 3a-d). Of particular interest is a line of prominent north-northeast trending magnetic highs (A5), located within the central part of the survey area. The A5 magnetic highs can be traced for about eight kilometers, from 64.51^∘^N/-16.54^∘^E to 64.54^∘^N/-16.50^∘^E, and show a one-kilometer right-lateral offset near 64.52^∘^N/-16.52^∘^E (Fig. 3a; see also Suppl. Mat. S3). However, the source of the A5 anomalies is concealed beneath the glacio-fluvial deposits.

### High-resolution THD magnetic anomalies

**Figure 4:**
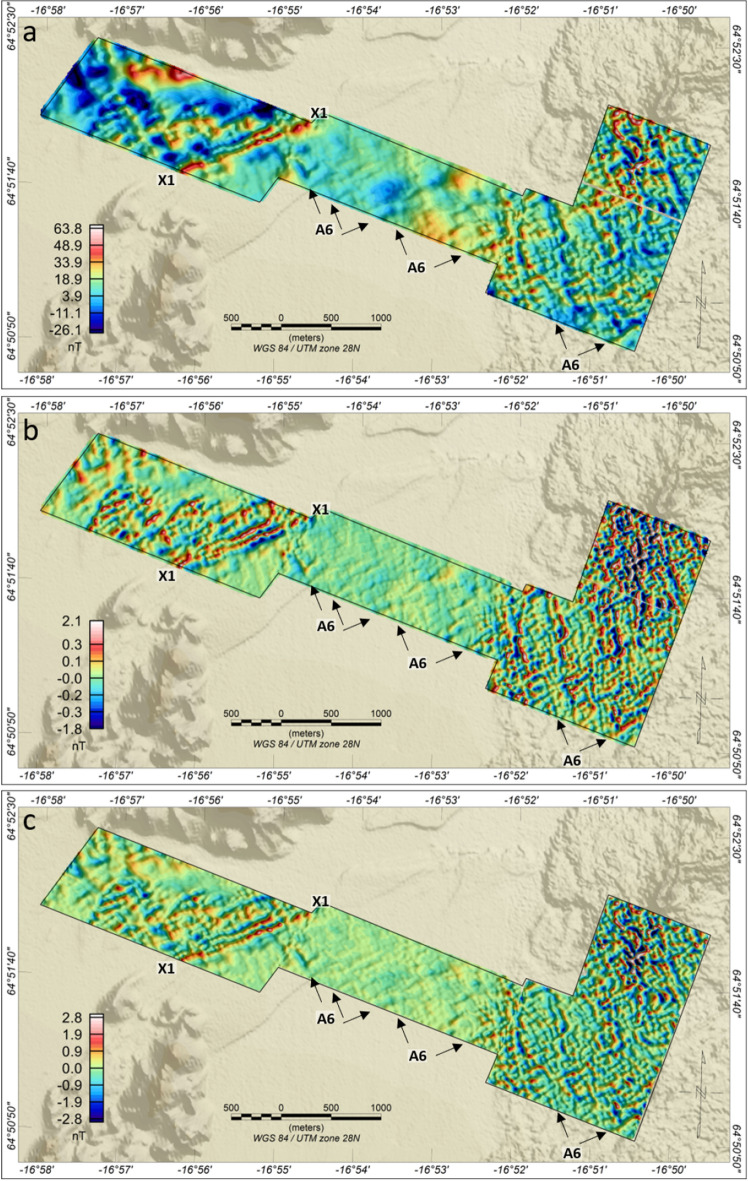
H200 Transverse Horizontal Difference (THD) magnetic data and derivative products. The magnetic data are displayed on top of digitized elevation data. (**a**) THD. (**b**) Along-Track difference of MHD (AD). (**c**) Discrete Vertical Difference of MHD (DVD). Labels refer to anomalies and structures discussed in detail. Full-scale plots of the subplots in Fig. 4 are found in Supplementary Material, Section S2.

**Figure 5:**
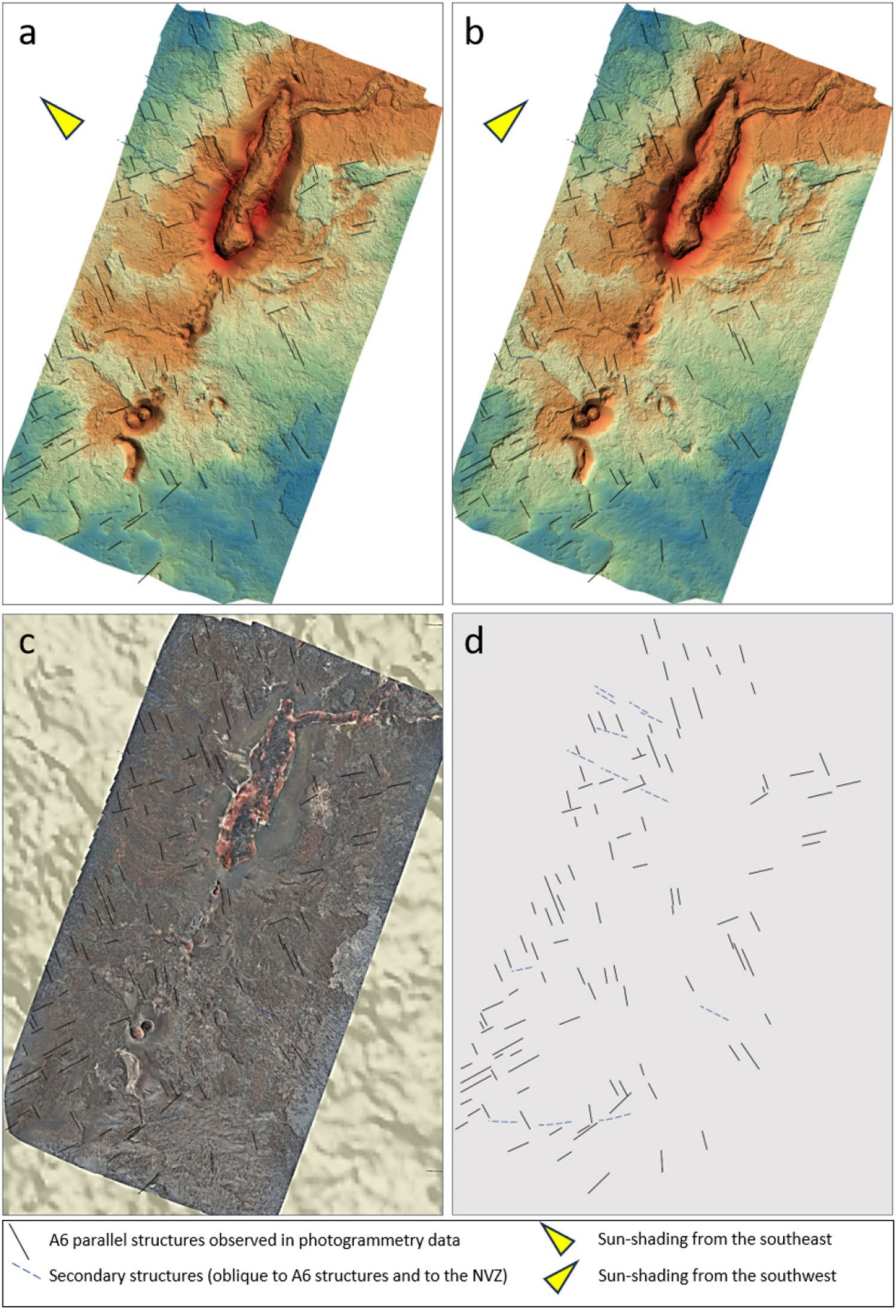
High-resolution drone photogrammetry data and structural analysis. (**a**) Sun-shading from the southeast. (**b**) Sun-shading from the southwest. (**b**) True colors. (**d**) Structures parallel to the A6 magnetic lineaments are observed in the photogrammetry data. See Suppl. Mat. S5 for full-page and non-interpreted photogrammetry images. Abbreviations: NVZ, Northern Volcanic Zone.

The high-resolution THD data (Fig. 4) reveals a pattern of short-wavelength, north-northwest (NNW), northwest (NW) and east-northeast (ENE) trending linear anomalies (A6) that are visible, both across the Holuhraun lava field and the glacio-fluvial outwash plain. The A6 anomalies intersect and offset the main north-northeast trending A2 and A5 anomalies (Suppl. Mat. S3). The low amplitude - yet short wavelength - character of the A6 anomalies across the outwash plain indicates an origin partly within the sediments in this area, i.e. the sediments appear to be weakly magnetic and heavily dissected by underlying basement faults. This interpretation is supported by magnetic property measurements of selected rock and sedimentary samples from the study area (see Suppl. Mat. S4). The A6 structures are not directly visible on the surface of the outwash plain, which is probably due to the frequent (daily) impact of sand storms and floods. However, high-resolution drone photogrammetry data across the Holuhraun main edifice show that the dense pattern of NNW-, NW- and ENE-trending A6 structures are evident in the basement (Fig. 5). Also, patterns of similar-trending structures are seen in the older topographic highs immediately to the northwest of the survey area (Suppl. Mat. S2 and S3). These observations indicate that the NNW-, NW- and ENE-trending A6 structures, visible in the high-resolution THD data (Fig. 4), constitute a dense pattern of secondary, pre-existing, faults that are continuously being reactivated.

**Figure 6:**
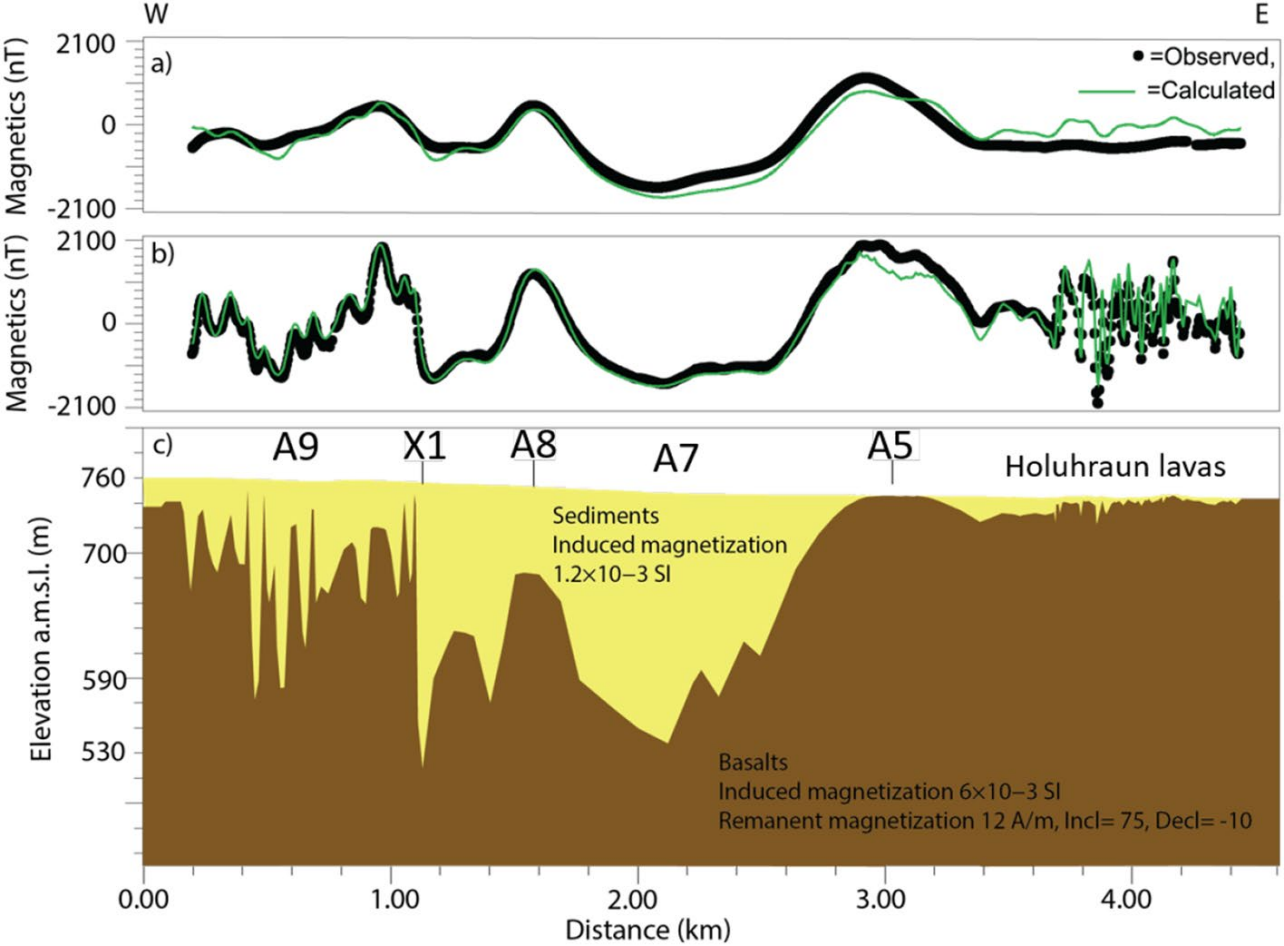
2D modeling (Oasis Montaj) along ground magnetic profile WG2. (**a**) Fit to drone magnetic data at 30m altitude. (**b**) Fit to ground magnetic data of WG2. (**c**) Geological model. We used a mean susceptibility and Natural Remanent Magnetization (NRM) intensity of the basement rock of 6x10^-3^ SI. A5, A7, A8, A9 and X1: Magnetic anomaly structures interpreted in Fig. 3.

**Figure 7:**
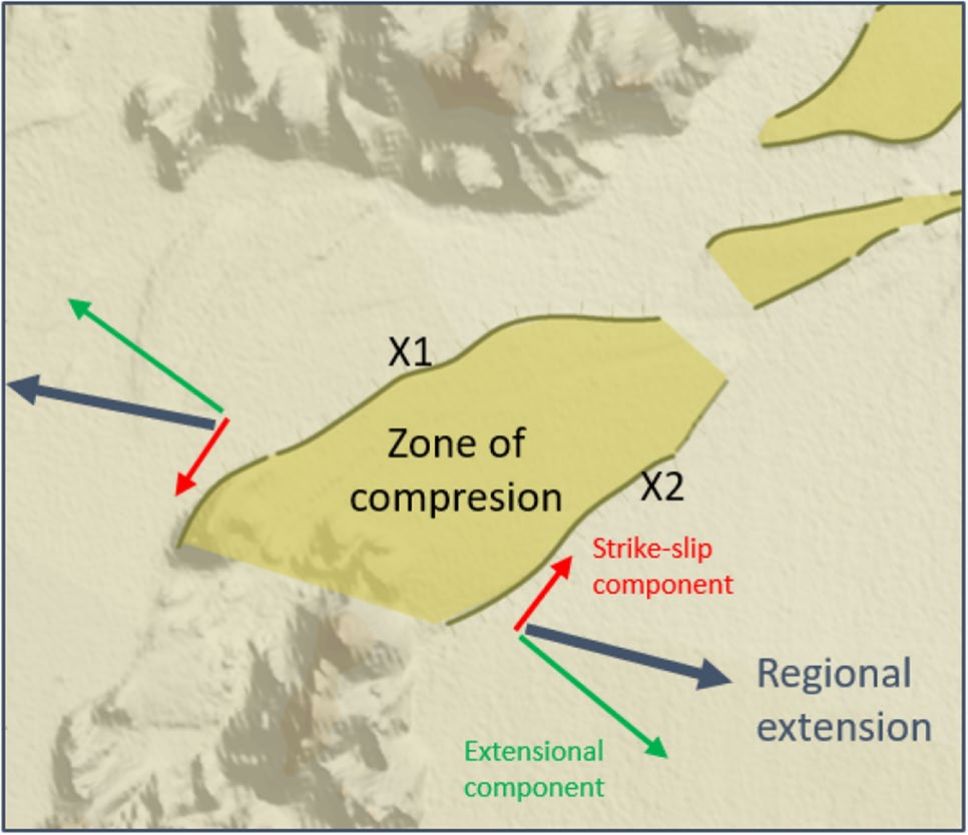
Simplified structural model for compression along the diagonal X1 and X2 structures as caused by strike-slip movements in relation to the regional extensional regime of the Northern Volcanic Zone (NVZ). The compression has caused an elevated topographic plateau between X1 and X2. See outline of area in Fig. S3 (Suppl. Mat.).

**Figure 8:**
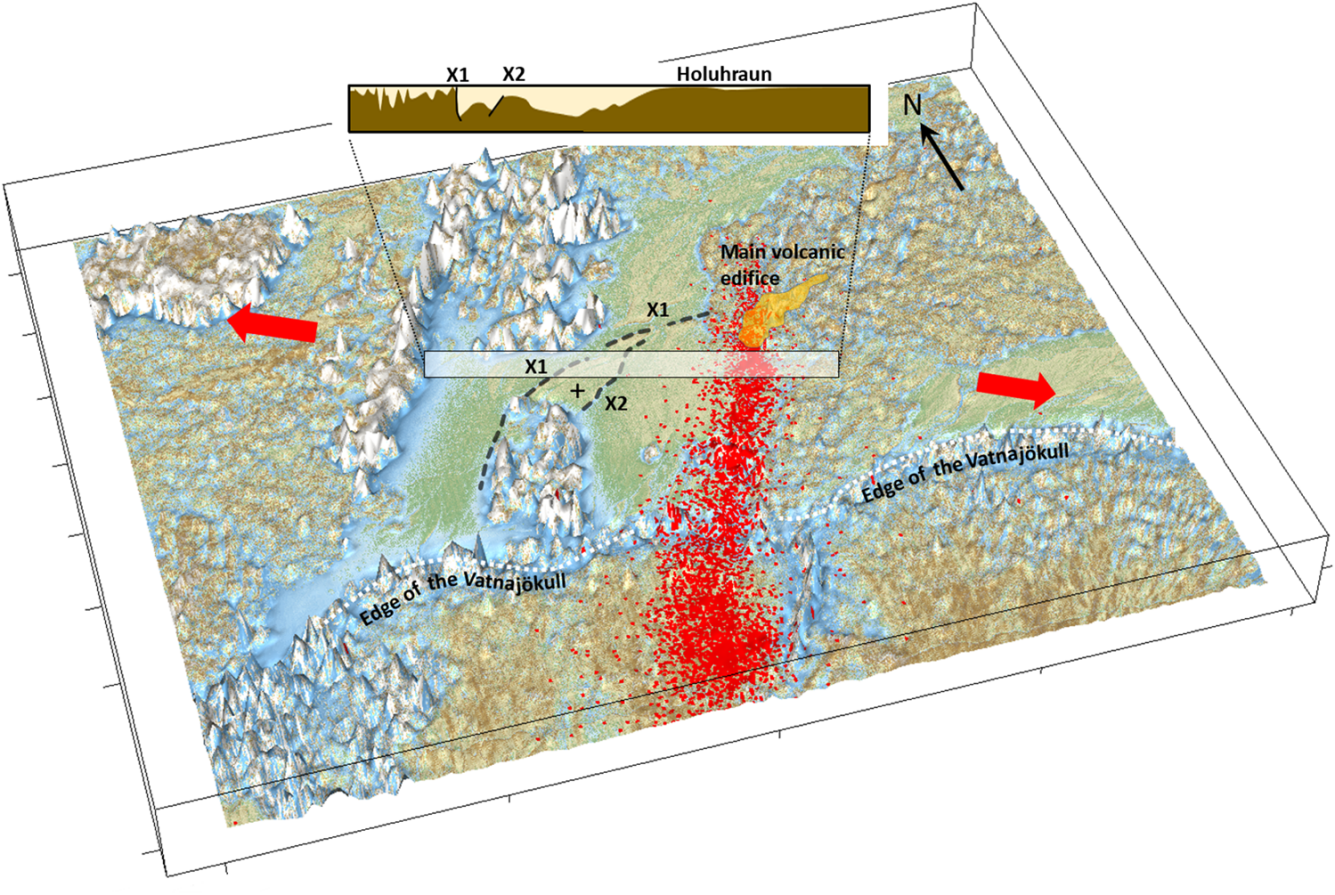
3D map view of the interpreted outline of the X1/X2 faults and the main Holuhraun volcanic edifice. Orange area: Outline of elevated volcanic edifice and the main lava run-off channel to the East-Northeast. ’+’: Sedimentary plateau defined by X1 and X2. Red dots: Earthquake locations in relation to the Holuhraun event and later. Red arrows: Regional extensional stress field. Background map: Derivative of digital topography data. The inset figure shows the interpreted basement-sediment interface along Profile WG2 (see Fig. 6).

The marked contrast in wavelengths between the glacio-fluvial outwash plain and the Holuhraun lava field is evident along the three ground magnetic profiles WG1–WG3 (Fig. 2b,c) but, particularly, along WG2 which transects the survey area in an east-west direction (Fig. 2b). Medium wavelengths are mapped across the sedimentary plain, while short wavelength, high-amplitude anomalies are observed across the Holuhraun lava field. We conducted a 2D magnetic modeling along profile WG2 to understand better the sources of anomalies beneath the sedimentary plain. The modeling was constrained by the magnetic property measurements (Suppl. Mat. S4) and adjusted to fit both the WG2 ground magnetic data as well as drone magnetic data at 30m altitude. The results (Fig. 6) indicate that the source of the broad anomaly A5 constitutes a shallow basement ridge that marks the transition between the Holuhraun lava field to the east and a 2-km-wide and >100-m-deep basin to the west. The basin is outlined by a broad NNE-trending magnetic low (A7) (Fig. 3a), which is intercepted in its western part by a north-northeast trending linear magnetic high (A8) modeled as a basement high (Fig. 6: A8). Immediately to the west of anomaly A8, the deep basin is modeled to terminate against a steep feature (Fig. 6: X1) that marks the transition to a shallow, highly irregular and faulted basement domain to the west. This basement domain generates a pattern of high-amplitude, short-to-medium wavelength, magnetic anomalies (A9), which are traceable in a north-northeast direction but also dissected by the secondary NNW-, NW- and ENE-trending A6 fault structures (Figs. 3, 4 and S4). The A9 faults and anomalies terminate abruptly to the southeast against the X1 structure, with some indications of shear deformation along X1.

The prominent X1 structure is associated with a distinct line of high-frequency, high-amplitude, THD anomalies, traceable in an east-northeast direction ($\sim$N060$E^\circ$) (Figs. 4 and S4). The steeply dipping nature of X1 (Fig. 6), its clockwise $\sim$45^∘^ rotation from the normal to the regional plate spreading direction of 104^∘^, and the indication of shear against the structure, suggest that X1 defines a prominent oblique-slip transform fault. Outside the high-resolution THD survey area, the signature of X1 becomes less distinct due to the lower resolution of the S100 surveys. However, east-northeast trending X1 anomalies and associated linear magnetic zones, defined by the termination and offset of north-northeast trending anomalies, are visible within the TMI derivatives to the edge of the Holuhraun lava field (Figs. 3b–d and S4).

### Surface expression of the X1 fault structure

On the surface, the X1 structure correlates closely with a sharp sedimentary escarpment X1S (Fig. S2) that displays a $\sim$2m vertical offset and separates a sedimentary plateau to the southeast from a depression to the northwest (Suppl. Mat. S2). The X1S escarpment is traceable as an east-northeast trending feature across the outwash plain to the north of the ground magnetic profile WG2. To the south of here, the sedimentary escarpment translates into a topographic boundary between a sedimentary outwash plain to the west and north-northeast trending topographic highs to the east (Suppl. Mat. S2).

Another linear magnetic anomaly pattern (X2), sub-parallel to X1 but with less amplitude, is visible about one kilometer to the south of X1 (Fig. 3). The east-northeast projection of the X2 structure correlates with the right-lateral offset of anomaly A5 (Figs. 3d and S4). The X2 anomalies also correlate with another sedimentary escarpment (X2S) (Fig. S3), which is located 0.2-1.0 km east of X1S and runs sub-parallel to X1S. The X1S and X2S escarpments together outline an elevated sedimentary plateau on the outwash plain (see Suppl. Mat. Figures S2c,d and S3). The correlation of X1S and X2S with the prominent basement structures X1 and X2, as mapped by the magnetic data, indicates that the sedimentary plateau may be a result of compression along X1 and X2 (Fig. 7). Of particular importance, the east to east-northeast extensions of the X1S and X2S escarpments correlate with the location of the main eruption site of the 1797 and 2014-15 Holuraun eruptions (Suppl. Mat. Fig. S3) as well as the distinct topographic east-northeast trend of the main lava drainage channel of the 2014-15 eruption in its northern end.

## Discussion

The path of a dike is governed by the pressure field produced by the overlying topographic load and the local stress field^12^. However, reactivation of pre-existing fractures is an important parameter for rifting mechanisms and dyke emplacements along divergent plate boundaries^13^. The northern dyke emplacement, graben, and eruptive fissures of the 2014-15 Holuhraun event, having a north-northeastern trend (25^∘^)^40^, are rotated 11^∘^ clockwise from the normal to the regional extensional stress field of 104^∘^^37^. The slight obliquity indicates that strike-slip accompanies the extensional opening along fault segments that are oblique to the N104^∘^E direction spreading direction^18,37,41^. Thus, evidence of left-lateral shear has been observed in relation to the north-northeast to northeast trending (N25^∘^E) Askja and Bardarbunga fissure swarms within the southern part of the Northern Volcanic Zone^13,18^. This obliquity of the Holuhraun emplacement structures to the regional stress field suggests a strong control from pre-existing structures on the eruptive event and/or influence from topographic loading^13,42^. Moreover, the 2014-15 event reoccupied the old 1797 Holuhraun craters^40^, which strengthens the interpretation of strong structural influence by pre-existing weaknesses within the rift zone.

The drone magnetic TMI and THD data in this study provide new insight into the complexity of the Bárðarbunga system - a remote and rather inaccessible part of the Icelandic rift system. By mapping subtle TMI and THD anomaly signatures, the new data provide high-resolution spatial information about primary and secondary structures of the rift zone and the correlation between surface/near-surface sedimentary structures (e.g., sedimentary escarpments and intra-sedimentary faults) and deeper basement structures beneath the glacio-fluvial plain north of the Vatnajökull ice cap. The TMI and THD data, particularly, reveal that the north-northeast trending dominant rift structures (N25^∘^E) are heavily intersected by dense patterns of secondary NNW-, NW- ($\sim$N15^∘^W-N25^∘^W) and ENE-trending structures ($\sim$N70^∘^E); these secondary structures are visible in both the sediments of the outwash plain and within the recent Holuhraun lava field (Figs. 4 and S4) as well as within the underlying basement (Fig. 5). The presence of these secondary structures suggests that they may also have played a secondary structural control in the recent volcanic eruption. Moreover, the identification of the prominent X1 structure and the correlation of X1 with an elevated sedimentary plateau that correlates to the east-northeast with the 2014–15 and previous Holuhraun main eruption sites suggest that the X1 structure enforced a primary structural control on the location of the principal eruptive fissure of both the Holuhraun eruptions. We, therefore, suggest that the northern-most lateral subsurface migration of lava during the Holuhraun events took place along north-northeast trending main rift structures until the lava, following a total of 48 km of sub-surface lateral migration, intersected with the east-northeast trending and older X1 structure at depth at its distal northeastern end, forcing forced lava to the surface (Fig. 8). Observations of similar persistent intrusive activity at ocean ridge-transform intersections and continental basement fault intersections are discussed by Mamaloukas-Fangoulis et al.^43^, Bowen and White^44^, and McCuaig and Hronsky^45^, respectively.

The lack of seismic activity along X1 (and X2) during the vertical magma migration event may indicate that the structure itself was not re-activated during the eruptive event but merely marks a significant lateral offset of north-northeast trending pre-existing weaknesses within the crust along which magma could migrate until forced to the surface. Alternatively, the vertical magma migration into shallow and pre-fractured crust was overall aseismic, creating little brittle failure. A similar interpretation has also been suggested for vertical magma migration along the north-northeast trending structures in the area^46^. However, our results indicate that the vertical migration of magma and final eruption along the same craters during the two Holuhraun events was not guided only by the laterally-migrating magma at depth reaching a topographic low and decrease in overburden pressure^12,46^ but also reached a prominent pre-existing diagonal structure that may have stalled further lateral migration.

## Methods

### Systems and survey specifications

The H200 magnetic THD surveys were collected using an in-house developed horizontal gradiometry bird (H200), which comprises two Optically Pumped magnetometers (OPMs), separated by a 2m across-track baseline and distanced from all onboard bird electronics by 2m. The two magnetometers are precisely positioned in 3D using an integrated centimeter-level NovAtel SPAN GNSS/INS positioning and attitude measurement system^36,47^. The S100 surveys were collected using an ultra-lightweight scalar single-sensor magnetometer bird (S100), equipped with one OPM^29^.

The H200 surveys were flown with a line spacing of 30m and a draped survey altitude of 30m (as taken from the magnetic sensors) above the terrain, while the S100 surveys were flown with a line spacing of 125m and 60m for the northern and southern parts, respectively, and a draped altitude of 40m above the terrain. Given the line spacing of the H200 flight lines, the H200 data have a Nyquist wavelength of 60m across track, while the corresponding Nyquist wavelength of the northern and southern S100 surveys is 250m and 120m, respectively. From the ±0.02nT fourth-order estimated dynamic noise level of the data (Fig. 3), the minimum amplitude anomalies detectable in the data is about 0.05nT peak-to-peak.

All drone surveys were collected with a west-northwest flight line orientation, chosen to cross-cut the dominant north-northeast strike of the Northern Volcanic Zone and, thereby, obtain an optimal correlation of magnetic anomalies between the survey lines. A mobile GSM-19W basestation from GemSystems was used throughout the campaign to monitor and correct for time-varying magnetic fields in addition to local geomagnetic observatory data. Three ground magnetic surveys were collected across the survey area using a GemSystems GSM-19gw backpack-mounted vertical difference system. Trimmed profile magnetic residual data from these ground surveys are shown in Fig. 1c,d.

### Data processing

We processed the S100 single-sensor data as well as single-sensor data from one of the H200 scalar magnetometers to total magnetic intensity (TMI) using standard processing steps^29^, including time-stamping and positioning, parallax correction, despiking, diurnal correction, correction of the main and super-regional magnetic field using the CHAOS X7 model^48^, line trimming, line levelling (without-ties)^49^, micro-levelling^50^, and reduction-to-pole^51^. Processing of the H200 THD data was performed using a recently developed equivalent source method^36,47^ that accounts for the unwanted attitude-induced responses of a gradiometry bird. An aerodynamically balanced magnetometer bird aligns to the relative airflow direction, which is a combination of ground speed and wind speed. Due to the relatively low survey speed of the survey drone during the campaign (10-11m/s) compared to wind speed (often 5-8m/s), the orientation of the drone-towed H200 bird system changed continuously throughout the surveys, i.e. the axes of the magnetic THD measurements changed continuously, causing unwanted attitude-induced responses. We utilized a radiometric equivalent source method^47^ to eliminate 3D attitude-induced responses in total field differences. The technique involves fitting a dipole surface to the measured data and then evaluating the response from this dipole surface at new pseudo-sensor locations unaffected by attitude-induced responses. The governing equation that relates the dipole surface to the measured data is given by

1 $$\begin{aligned} d_j&= -\frac{1}{4 \pi } \sum _i^M \mu _i \Omega _{i,j} \end{aligned}$$

where $\mu$ is the gradient of the surface magnetic polarisation in the direction of the ambient field, *d* is the measured total field data, and $\Omega$ is the solid angle subtended by the facets from the data points.

To fully construct the forward problem of the gradiometric equivalent source method, we need to relate the dipole surface to the total field differences. To do this, we subtract Equation 1 by itself with $\Omega$ calculated separately for each sensor location. This subtraction can be written in matrix form as

2 $$\begin{aligned} \Delta \varvec{d}&= -\frac{1}{4\pi } (\varvec{\Omega }_{S1} - \varvec{\Omega }_{S2}) \varvec{\mu } \end{aligned}$$

3 $$\begin{aligned}&= \varvec{G} \varvec{\mu } \end{aligned}$$

where $\varvec{\Omega }_{1}$ and $\varvec{\Omega }_{2}$ are the matrices containing the solid angles from each sensor. $\Delta \varvec{d}$ is the scalar field differences. Now the method involves solving the inverse problem of Equation 2 with a smoothing constrict and a forward evaluation of the resulting dipole surface to the new pseudo sensor position. The total field differences should now be free from striping and high-frequency noise in the total field differences typical for attitude-induced responses^36,47^.

Finally, well-established numerical methods were applied to the TMI single-sensor data and the THD gradiometry data to enhance short-wavelength anomaly signatures. These methods include the Discrete Vertical Derivative (DVD)^51^, the Along-track Derivative (AD)^52^, and the Tilt Derivative (TD)^51^. All processing and data enhancement was done using in-house developed software.

### Supplementary Information

Supplementary Information.

## Data Availability

The S100 and H200 datasets used during the current study are available from the corresponding author. Please send an email to ards@space.dtu.dk.
