# Supplementary material for: Pre-existing structural control on the recent Holuhraun eruptions along the Bárðarbunga spreading center, Iceland
Source: Sci Rep. 2024 Feb 10;14:3399. doi: 10.1038/s41598-024-53790-x (PMC10858284; doi:10.1038/s41598-024-53790-x)
Supplement: Supplementary file 1 — Supplementary Information. [file 41598_2024_53790_MOESM1_ESM.docx]

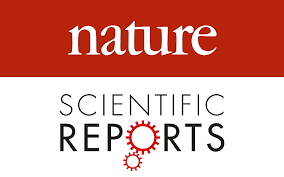


Supporting Information for

**Pre-existing structural control on the recent Holuhraun eruptions along the Bárðarbunga spreading center, Iceland**

Arne Døssing^1^, Mick E. Kolster^1^, Eduardo L. S. da Silva^1,5^, Adrian R. Muxworthy^3,4^, Jacob
Thejll Petersen^1^, Morten S. Riishuus^2^

1 Crustal Magnetometry Technology & Research Group (CMAGTRES), Division of Geomagnetism & Geospace, DTU Space, Centrifugevej 356, 2850 Kgs. Lyngby, Denmark.
2 Faroes Geological Survey, 34 Jóannesar Paturssonar gøta, Tórshavn 100, Faroe Islands.
3 Department of Earth Science and Engineering, Imperial College London, South Kensington Campus, London SW7 2AZ, UK.
4 Department of Earth Sciences, University College London, Gower Street, London, WC1E 6BT, UK

5 UMag Solutions Aps, Nørgaardsvej 26, 2800 Lyngby, Denmark

**Contents of this file**

Text S1-S6

Figures S1 to S10

Tables S1 to S2

**Introduction**

The Supporting Material document contains six sections that each contain several figures and/or tables. In Section S1, we describe details about the data collection. In Section S2, we provide a detailed structural lineament analysis of publicly available topographic data, while Section S3 highlights our interpreted magnetic lineaments. Section S4 provides details about rock magnetic properties as measured on samples collected as part of the study. Section S5 shows the results of our drone photogrammetry data and evidence of fine-scale complex structures in the basement – structures that are also found in the high-resolution THD magnetic data. Finally, Section S6 shows details of our 2D magnetic modeling along one of the long ground magnetic profiles across the glacio-fluvial plain.

S1: Data collection - additional details

The base camp for the field campaign was set up at Dreki near the eastern flank of the Askja volcano (Figure S1). From here, daily transport to the survey location near Holuhraun could be reached within two hours on 30 km of dirt roads. Magnetic data collected during the campaign are listed in Table S1. Due to below-zero temperatures and outside storage facilities, the survey magnetometers generally failed to start up during the first week of the campaign. Combined with windy weather, multiple days were encountered with limited (or no) data collection.


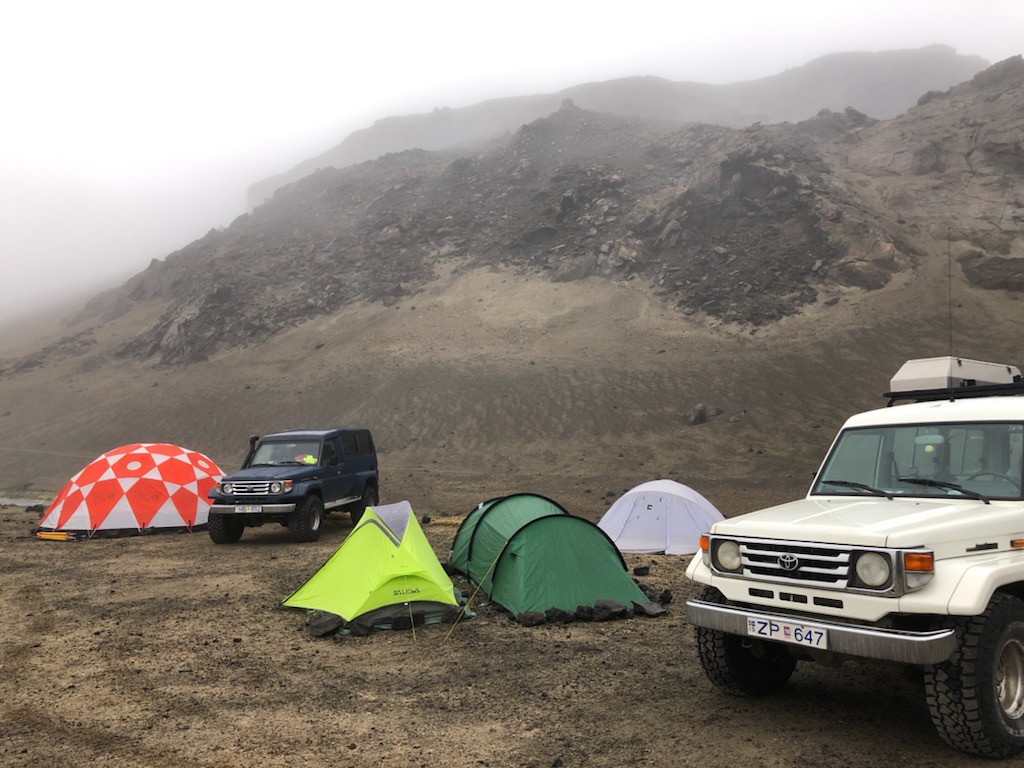


**Figure S1.** Base camp near Dreki on the eastern flank of the Askja central volcano. All survey equipment was stored in tents overnight.

| Date | System | Survey | Line spacing (m) | Altitude A.G.L. (m) | Line-km |
| --- | --- | --- | --- | --- | --- |
| 20190818 | H200 | C3 | 30 | 30 | 50 |
| 20190818 | S100 | N1 | 125 | 40 | 12 |
| 20190819 | H200 | C2 | 30 | 30 | 75 |
| 20190819 | S100 | N1 | 125 | 40 | 60 |
| 20190819 | S100 | N2 | 125 | 40 | 17 |
| 20190820 | S100 | N2 | 125 | 40 | 28 |
| 20190823 | H200 | C1 | 30 | 30 | 38 |
| 20190823 | S100 | N2 | 125 | 40 | 48 |
| 20190823 | S100 | N3 | 60 | 40 | 65 |
| 20190824 | H200 | C3 | 30 | 30 | 48 |
| 20190824 | S100 | N4 | 60 | 40 | 47 |
| 20190825 | H200 | C3 | 30 | 30 | 38 |
| Total |  |  |  |  | ~526 |

**Table S1.** Survey acquisition parameters of the drone magnetic data.

In addition to the magnetic data acquisition (Table S1) we also collected two drone photogrammetry surveys across the 2014 Holuhraun eruption site, and seven rock samples were collected for rock magnetic analysis.

Two orthophotos and digital surface models were recorded by means of two photogrammetry surveys to enable terrain following magnetic surveys and to track surface features and their continuity to subsurface features mapped with magnetic sensors. A total of 1068 images were collected with a lightweight Parrot Anafi UAV between 850 and 900 meters above mean sea level covering a total of 1.9 km^2^. Ground control points (GCPs) were not used in either of the areas as the rough and dangerous terrain within the survey areas prevented the team to safely distributing the GCPs. Additionally, the accuracy that would result from the addition of the GCPs is negligible compared to the subject features' dimensions.

Processing of the photogrammetry data was performed using PIX4D software by means of the structure-from-motion approach (Westoby et al., 2012), which used the overlapping images of the ground from the same camera at different positions to automatically triangulate and fit a point cloud that is subsequently used to correct and stitch the orthophotos.

**S2: Topographic analysis**

The highlands surrounding the glaciofluvial plain between the Dyngvøjökull and Askja volcanoes display densely packed linear structures with a dominating NNE-trend (25^o^E), i.e. parallel to the trend of the NVZ (see Figure 1 in Main article). Many of these structures are associated with abrupt changes in elevation, indicating a vertical offset along the structures. However, secondary structures, having a NW- to NNW-trend (300-330^o^E) and an ENE-trend (60-65^o^E), are also traceable in the elevated topography to the west of the survey area. This is evident in the topographic data displayed in Figure S2). In contrast, the newly formed lava fields of the Holuhraun eruptions display only a few prominent linear structures of significant offset.

In the sedimentary lowlands, located between the highs to the west and the lava fields to the east, the glaciofluvial plain display two prominent escarpments (see Figure S2), which are highlighted in "residual topography data" (Figure S2b) and in topography data displayed with different linear color scales (Figure S2c,d).

Figure S3 displays a summary of the topographic features interpreted within the drone survey area and its immediate surroundings based on publicly available Digital Elevation Data. Please notice that the northeastern termination of the sedimentary escarpments correlates with the northern edge of the 2014 Holuhraun eruption and that the trend of these escarpments is parallel to the ENE-trend of the main outflow from the eruption site.


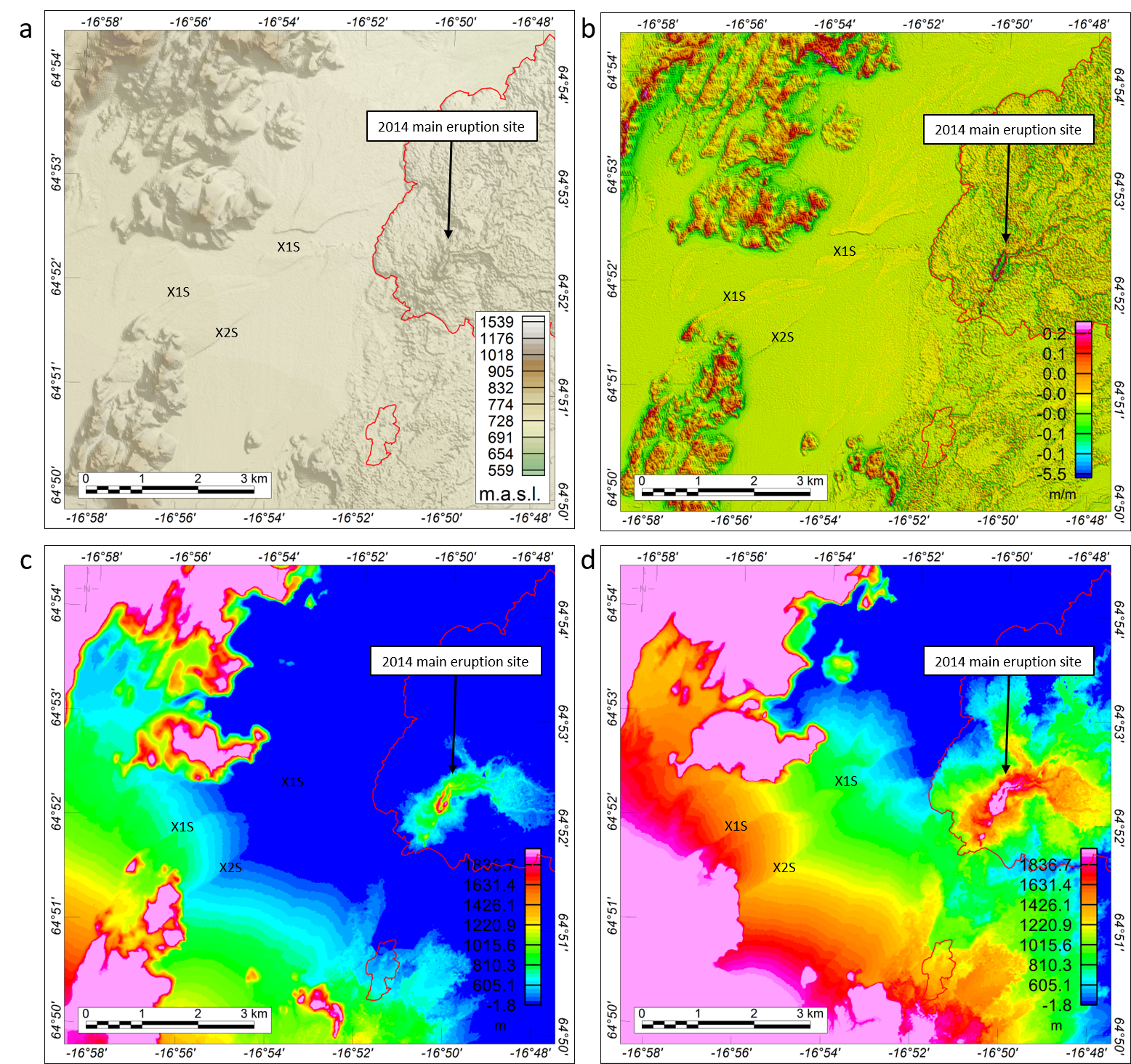


Figure S2. Topographic trends highlighted by various enhancement methods. (a) Topography with normal shadow display. (b) Vertical derivative of the topography. (c) and (d) Topography displayed with different linear color scales to highlight small elevation changes related to the glacio-fluvial outwash plain and the Holuhraun 2014 main eruption edifice. Red polygons: Outline of 2014 event.


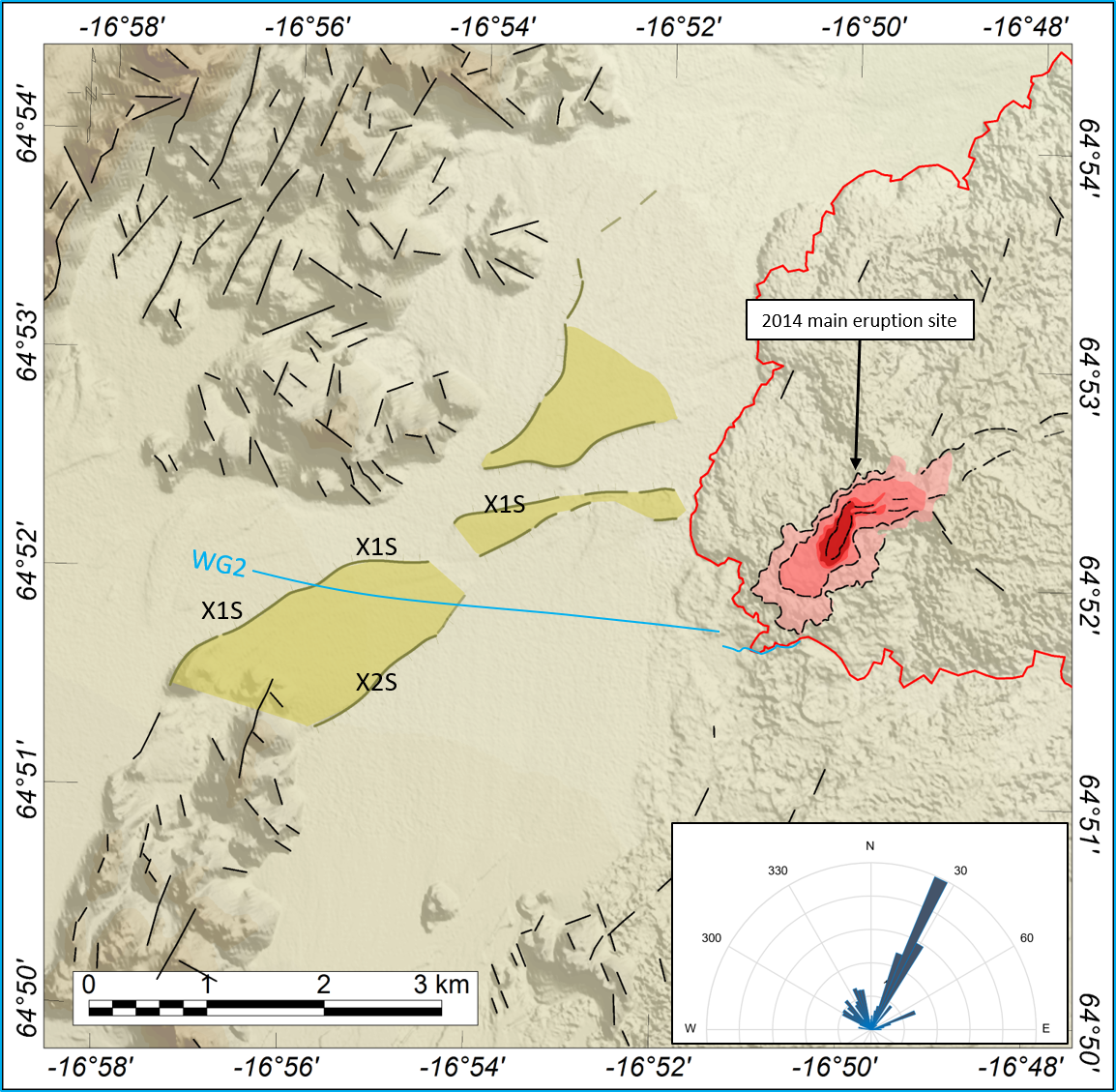


**Figure S3.** Visible topographic lineaments and sedimentary escarpments within and around the survey area. Yellow transparent areas: Elevated sedimentary topography bounded by escarpments. Red transparent areas: Topographic outline of the 2014 eruption edifice. Notice the sharp ENE-bend in the topography related to the main lava outflow from the eruption edifice. Red polygons: Outline of 2014 event. Light blue line: WG2 ground magnetic profile location. Inset figure: Azimuth of visible topographic lineaments (this study), weighted according to the traceable length of each lineament.

## S3: Interpreted magnetic lineaments


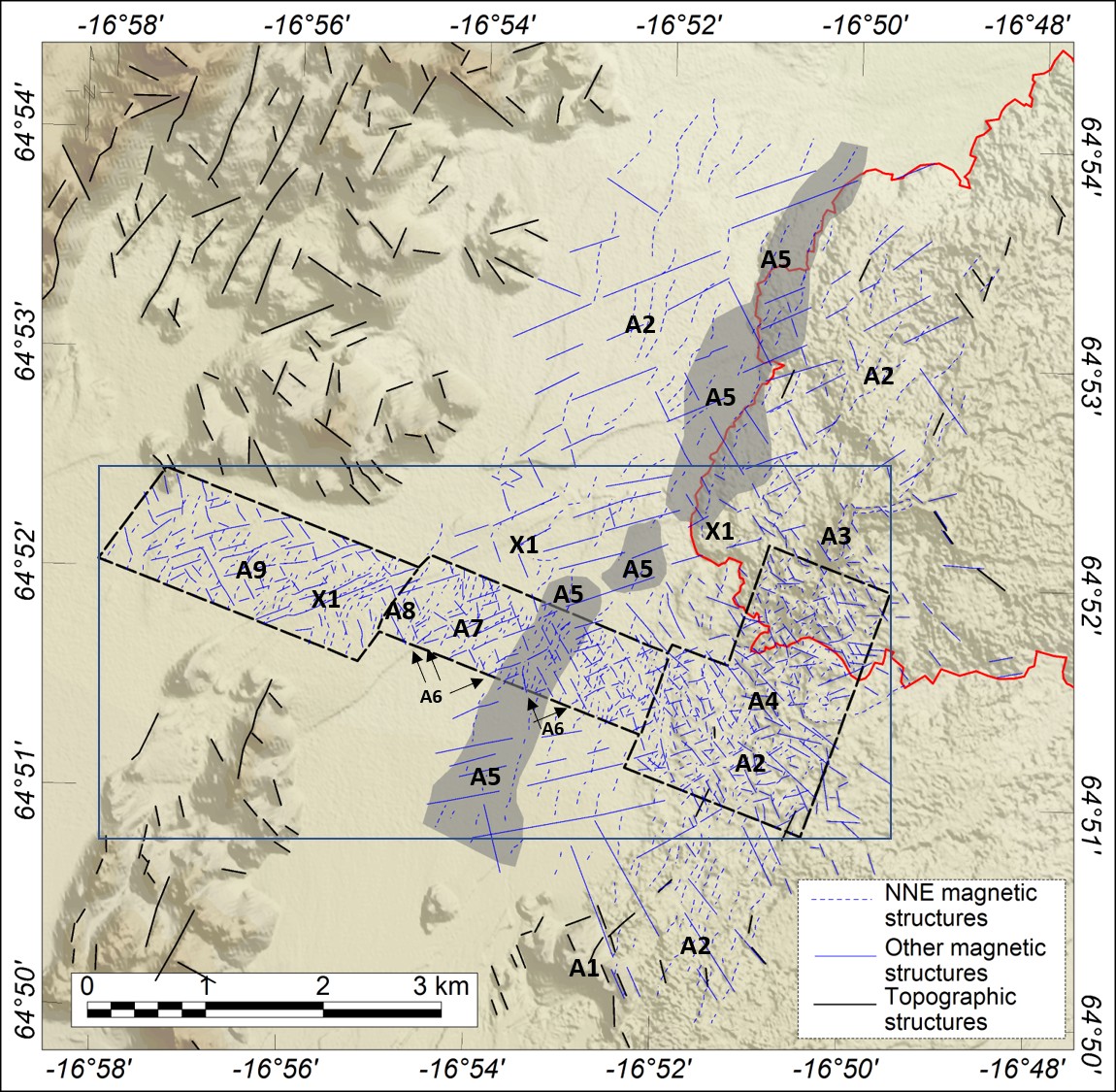


**Figure S4.** Interpreted magnetic lineaments based on the high-resolution H200 and medium-resolution S100 data. The magnetic lineaments are depicted together with topographic lineaments, visible in the elevation data (see Suppl. Mat. S2). Red line: Outline of 2014 Holuhraun lava.


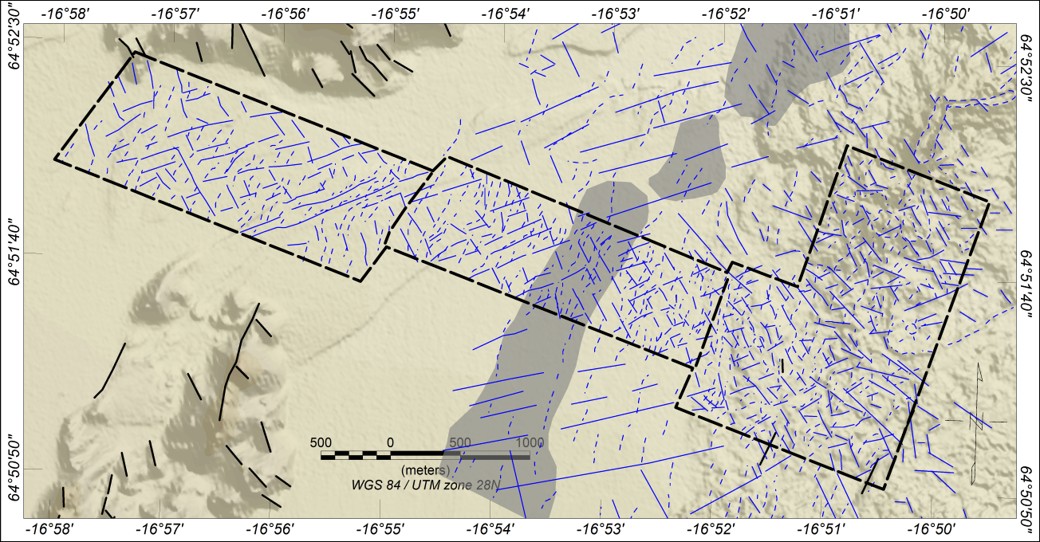


**Figure S5.** Magnetic lineaments interpreted from the H200 THD data.

## S4: Rock magnetic properties

Seven rock samples and one sediment sample were collected. The locations of the samples taken are shown in Figure S6.


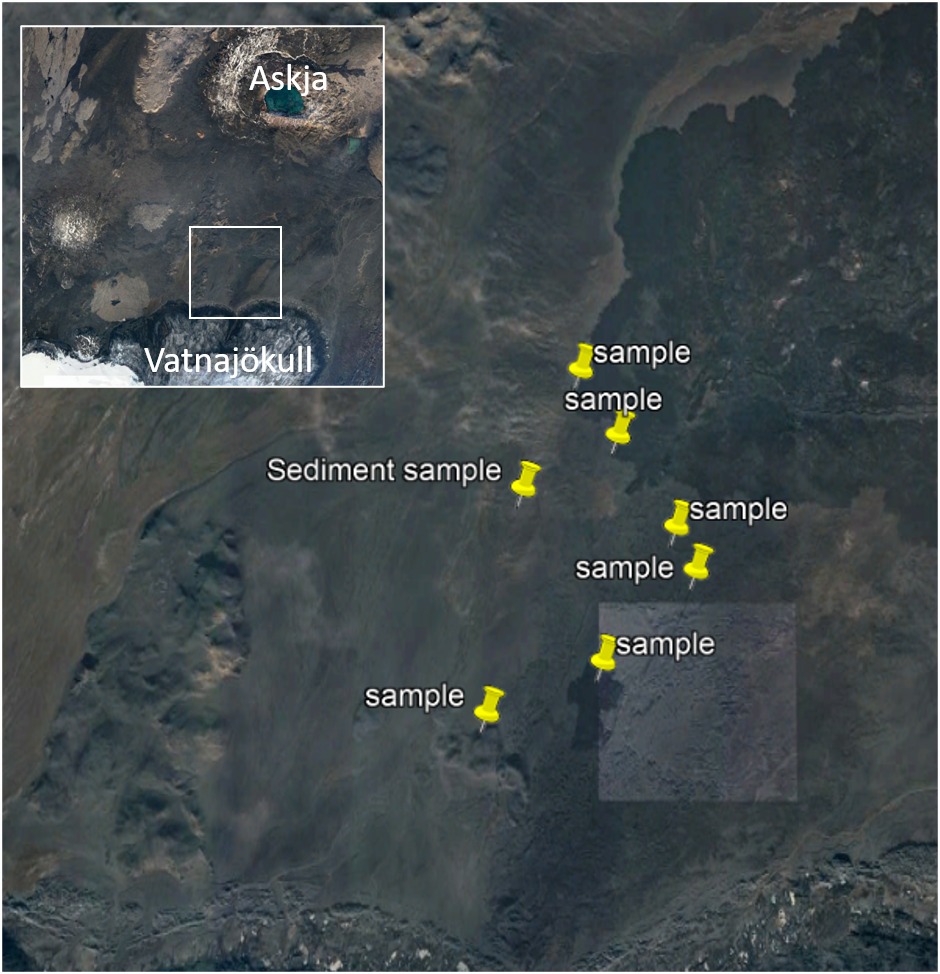


Figure S6. Location of hand samples for rock magnetic analysis. “sample” refer to hard rock samples that were oriented.

From the seven rock samples, we extracted between two and four oriented 1 cm sub-cores. From these, the mean bulk volume magnetic susceptibility was determined for each hand sample using a Bartington MS2 meter. This was done by measuring each sub-core three times, and taking the arithmetic mean for each hand sample. The Natural Remanent Magnetization (NRM) information of each oriented sub-core was measured from an average of three measurements per sub-core, using an AGICO JR5A magnetometer at the Paleomagnetic laboratory of Imperial College London. The NRM intensities were averaged using the procedure as the magnetic susceptibilities. The NRM directions were generally very consistent; however, for the cases where only two sub-cores were used to determine the mean NRM direction, the α95 was high (~60˚) due to the low number of samples. Where four sub-cores were used the α95 was as low as 3˚.

For the sediment sample (Figure S7), only magnetic susceptibility was measured and subsequently averaged taking the arithmetic mean.


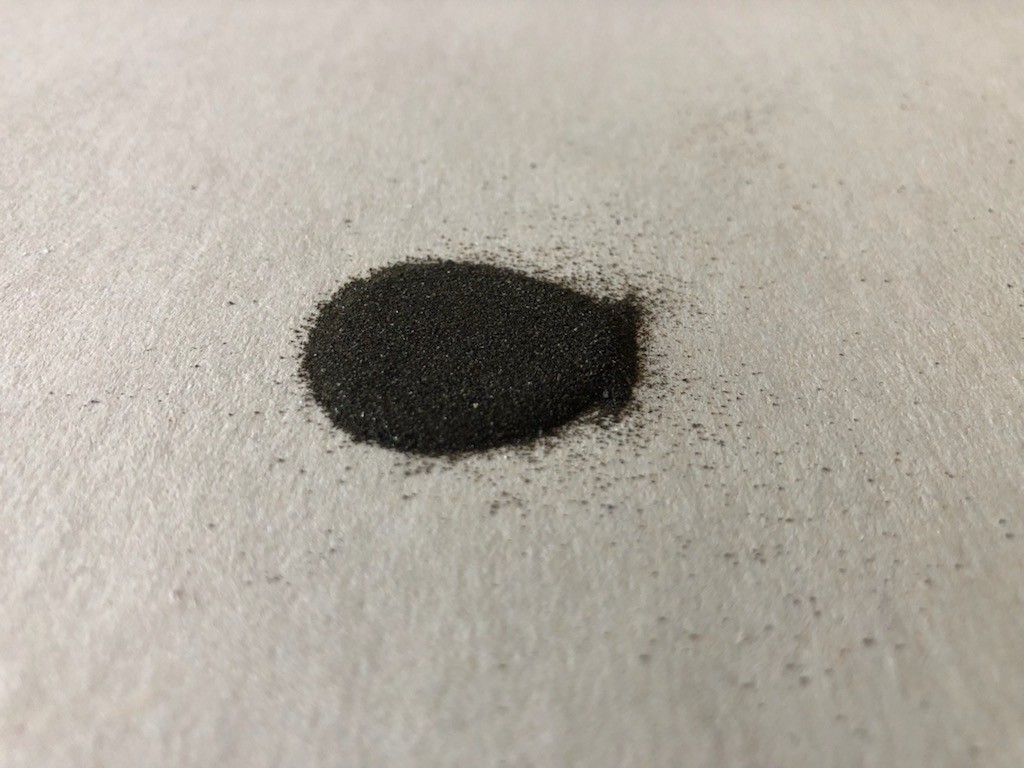
The results of the rock magnetic measurements are shown in Table S2.

**Figure S7.** Picture of a sand sample from the Jökulsá á Fjöllum river glacio-fluvial outwash plain.

| Sample | Cores | Speci. | Mean *x* | Std | Mean NRM | Std | Mean Dec | Mean Inc | *a*95 | Dip | Strike |
| --- | --- | --- | --- | --- | --- | --- | --- | --- | --- | --- | --- |
| 1 | 2 | 2 | 555 | 90 | 6.1 | 1.9 | 356 | 66 | 72 | 19 | 239 |
| 2 | 2 | 2 | 515 | 88 | 8.2 | 1.8 | 14 | 71 | 37 | 48 | 34 |
| 3 | 2 | 2 | 767 | 124 | 38 | 10 | 252 | 70 | 21 | 40 | 98 |
| 4 | 1 | 3 | 314 | 15 | 5 | 0.7 | 91 | -65 | 3 | 75 | 79 |
| 6 | 1 | 4 | 1085 | 92 | 14.9 | 1.8 | 210 | 65 | 3 | 88 | 104 |
| 7 | 2 | 2 | 537 | 50 | 5.4 | 2 | 231 | 75 | 61 | 72 | 146 |
| Sedim. | NA | 1 | 120 | NA | NA | NA | NA | NA | NA | NA | NA |

**Table S2.** Magnetic properties of rock and sedimentary samples. Unit of the mean susceptibility (Mean *x*) is x10^−5^ SI. Unit of the mean NRM intensity (Mean NRM) is A/m. The Mean Dec, Mean Inc, *a*95, Dip and Strike are all in degrees. All rock magnetic measurements were carried out at the Natural Magnetism Group Laboratory at Imperial College, London.

## S5: Drone photogrammetry data across the Holuhraun 2014 main edifice

**
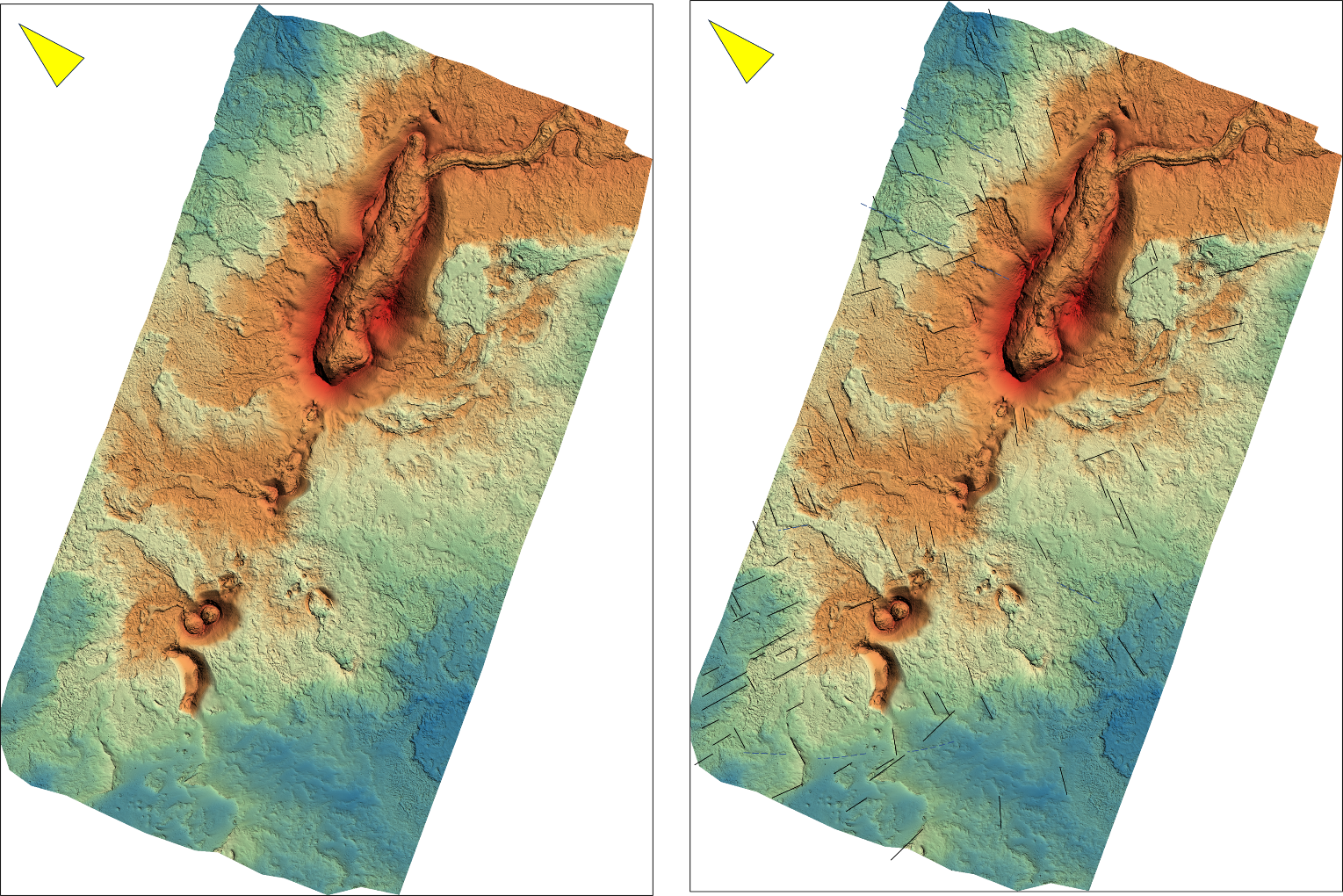
**

**Figure S8.** Drone photogrammetry image (un-interpreted) of the Holuhraun main edifice. Southeast sun-shading (yellow arrow in the upper left corner) and false coloring. An interpretation of the visible structures is shown in Figure 5 (Main article).


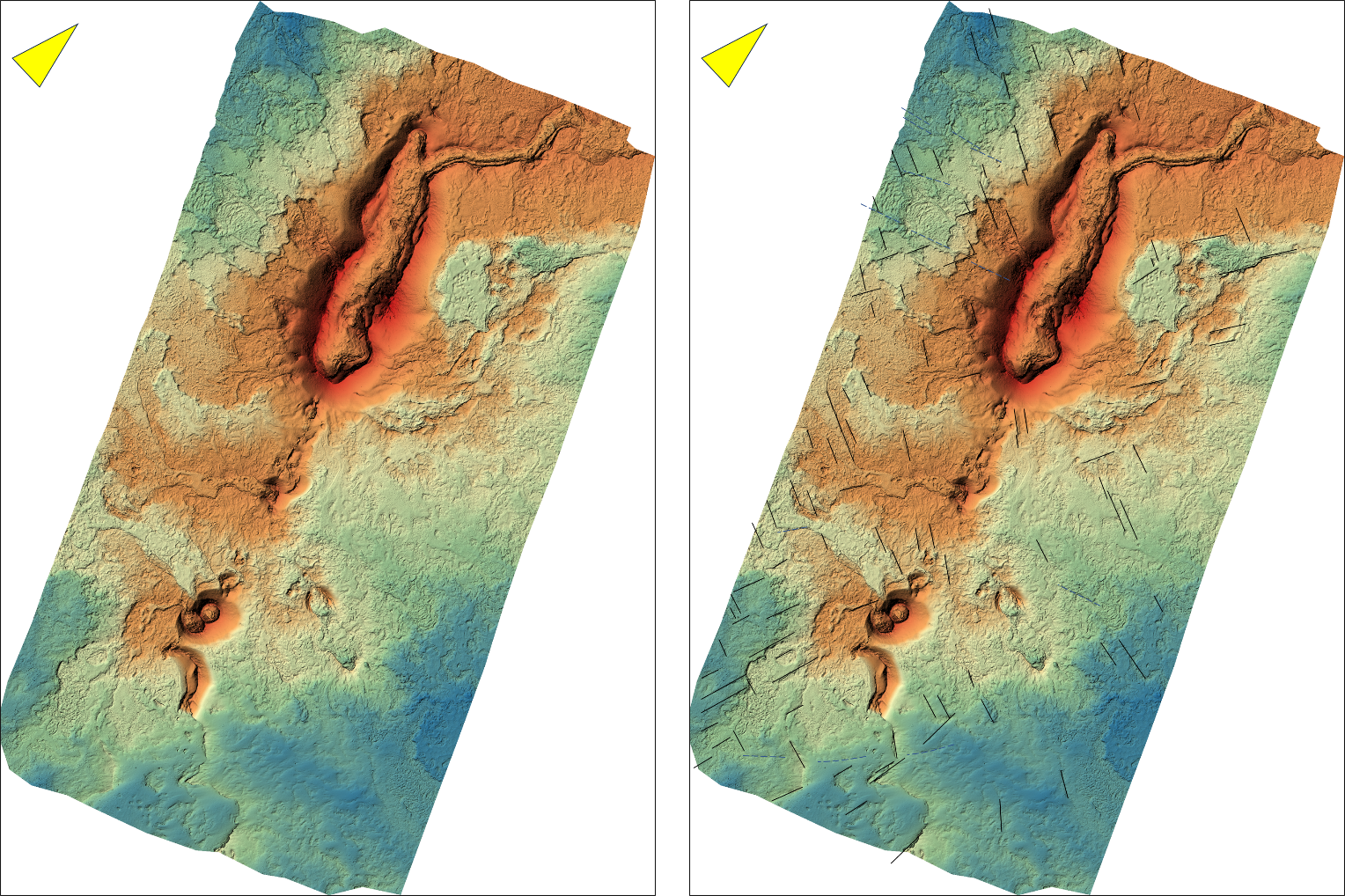


**Figure S9.** Drone photogrammetry image (un-interpreted) of the Holuhraun main edifice. Southwest sun-shading (yellow arrow in the upper left corner) and false coloring. An interpretation of the visible structures is shown in Figure 5 (Main article).


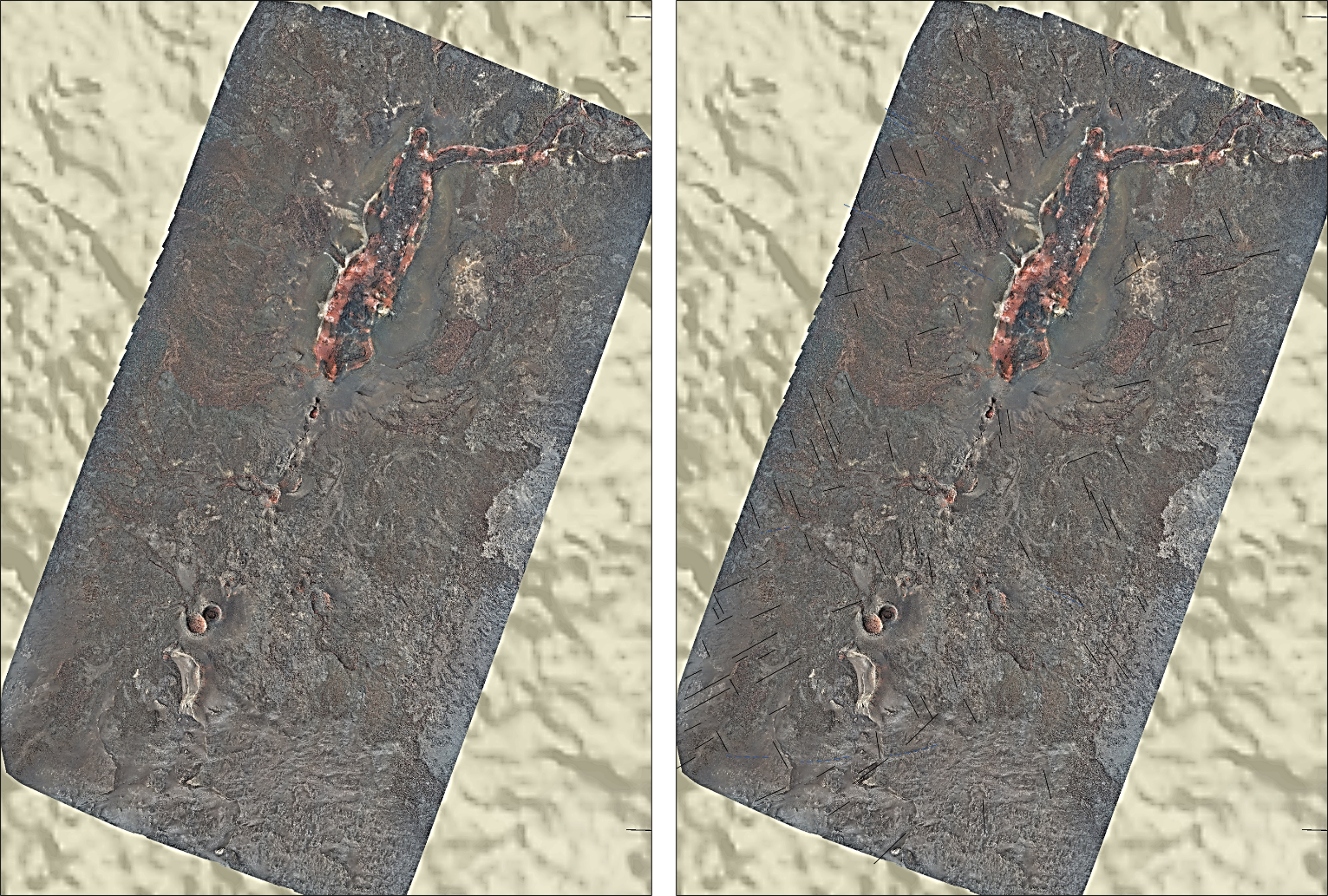


**Figure S10.** Drone photogrammetry image (un-interpreted) of the Holuhraun main edifice. True color. An interpretation of the visible structures is shown in Figure 5 (Main article).

***References (Supporting Material)***

Westoby, M. J., Brasington, J., Glasser, N. F., Hambrey, M. J., & Reynolds, J. M. (2012). ‘Structure-from-motion’ photogrammetry: A low-cost, effective tool for geoscience

applications. *Geomorphology*, *179*, 300–314.
